# Relationship between Outer Retinal Layers Thickness and Visual Acuity in Diabetic Macular Edema

**DOI:** 10.1155/2015/981471

**Published:** 2015-06-08

**Authors:** Raymond L. M. Wong, Jacky W. Y. Lee, Gordon S. K. Yau, Ian Y. H. Wong

**Affiliations:** ^1^Department of Ophthalmology, The University of Hong Kong, Room 301, Level 3, Block B, Cyberport 4, Pokfulam, Hong Kong; ^2^Department of Ophthalmology and Visual Science, The Chinese University of Hong Kong, Kowloon, Hong Kong; ^3^Department of Ophthalmology, Caritas Medical Centre, Kowloon, Hong Kong

## Abstract

*Purpose*. To investigate the correlation of outer retinal layers (ORL) thickness and visual acuity (VA) in patients with diabetic macular edema (DME). *Methods*. Consecutive DME patients seen at the Retina Clinic of The University of Hong Kong were recruited for OCT assessment. The ORL thickness was defined as the distance between external limiting membrane (ELM) and retinal pigment epithelium (RPE) at the foveal center. The correlation between total retinal thickness, ORL thickness, and vision was calculated. *Results*. 78 patients with DME were recruited. The mean age was 58.1 years (±11.5 years) and their mean visual acuity measured with Snellen chart was 0.51 (±0.18). The correlation coefficient between total retinal thickness and visual acuity was 0.34 (*P* < 0.001) whereas the correlation coefficient was 0.65 between ORL thickness and visual acuity (*P* < 0.001). *Conclusion*. ORL thickness correlates better with vision than the total retinal thickness. It is a novel OCT parameter in the assessment of DME. Moreover, it could be a potential long term visual prognostic factor for patients with DME.

## 1. Introduction

Diabetes mellitus is one of the commonest chronic diseases affecting all populations especially developed countries. Diabetic macular edema (DME), being a complication of diabetes, is an important cause of visual loss in developed countries [1, 2]. Treatment of diabetic macular edema is readily available and management guidelines of diabetic macular edema have largely evolved around the use of new laser machines, newer pharmacological agents such as antivascular endothelial growth factors (anti-VEGF), and different steroid preparations [3–7]. In the past decade, the evaluation of treatment efficacy was mainly based on visual acuity measurements and the detection of structural improvement on optical coherence tomography (OCT) scans. Undoubtedly, the fast, objective, and noninvasive OCT has emerged into a valuable tool, not only in DME, but also in other macular diseases such as age-related macular degeneration (AMD) and central serous chorioretinopathy. However, the correlation between OCT measured variables and visual acuity has not been well established. Although reports have shown good correlation of OCT measured macular changes with vision, there were also reports that produced contradicting results [8]. A comprehensive understanding of the various OCT measured parameters in DME and its clinical implications is yet to be determined.

The advancement in optical coherence tomography (OCT) technologies including the increase in speed of scanning and higher axial resolution (up to ~3 microns for certain OCT machines) has made visualization of the retinal microstructures possible [9–11]. Reports have looked into the morphological changes happening in the outer retinal hyperreflective bands in subjects with various retinal diseases. The integrity of the inner segment/outer segment (IS/OS) junction has been found to correlate well with visual acuity in subjects with retinal conditions such as retinitis pigmentosa and postmacular hole operation [12, 13]. The length of the photoreceptor outer segment (PROS) has also been reported to be able to predict visual acuity in DME more accurately than the more commonly used macular thickness [14]. Another important retinal segmentation noticeable on spectral- (Fourier-) domain OCT, the external limiting membrane (ELM), and its correlation with visual acuity in diabetic macular edema has not been well studied. Being situated between the cell nucleus and inner segments of photoreceptors, ELM may also be a possible OCT based parameter to be used indirectly in the assessment of photoreceptor functions. The aim of this study was to find out the correlation between visual acuity and the distance between the ELM and the retinal pigment epithelium (RPE), as a novel parameter in the assessment of DME.

## 2. Methods

### 2.1. Study Subjects

Consecutive patients with DME seen at the Eye Clinic of The University of Hong Kong over a 3-month period were recruited for this study after informed consent. Inclusion criteria were diabetic patients aged 18 or above and capable of giving consent and having DME as evidenced clinically with a slit lamp biomicroscopy or on OCT scans. Major exclusion criteria included poor media clarity that would affect vision and hinder satisfactory OCT image acquisition and presence of conditions other than DME that would affect macular thickness which were also excluded, such as age-related macular degeneration, vitreomacular traction, epiretinal membrane, full thickness or lamellar macular holes, and other causes of macular edema such as retinal venous occlusion. Eyes with subretinal fluid at fovea were excluded as well because their presence would result in falsely high measurement of outer retinal layers thickness (ORL thickness), since the distance is defined in this study as the distance between ELM and RPE. Baseline demographics of patients were collected. Best-corrected visual acuity (BCVA) was measured with Snellen's visual acuity charts. A comprehensive ocular examination including dilated fundal slit lamp biomicroscopy and macular OCT scan were performed. If both of patient's eyes were eligible for recruitment, only the right eye was used in the data analysis. This study has been approved by the Institutional Review Board of The University of Hong Kong and was performed in accordance with the Declaration of Helsinki.

### 2.2. Optical Coherence Tomography

Scanning with the Spectralis HRA + OCT system (version 3.2.1.0, Heidelberg Engineering, Inc., Heidelberg, Germany) was performed using the built-in 7-line raster scan protocol. Images were averaged from 100 frames for the purpose of noise reduction. The OCT scans were excluded if the image quality was less than 30 decibels. All study eyes were dilated with mydriatic eye drops before image acquisition. Patients were instructed to fixate on the intrinsic fixation target during the whole process of OCT scanning. If the patient was not fixating well and the center of image was not on center of the fovea, manual adjustment was performed. The OCT scans were performed by a single experienced optometrist.

### 2.3. Determination of Outer Retinal Layer (ORL) Thickness

The Spectralis sd-OCT data were analyzed on the Heidelberg Explorer by a single retina specialist. The horizontal line scans crossing foveal centers of patients were chosen for analysis. The internal limiting membrane (ILM), ELM, and RPE segmentation of retina were manually set. When the hyperreflective layers were identified, the point of maximal brightness of each band was chosen to be the locations of the corresponding ELM and RPE bands. The central foveal point thickness was defined as the distance between the ILM and RPE at the foveal center whereas the ORL thickness was the distance between ELM and RPE at the foveal center. Patients with disruption of ELM or RPE segmentation on OCT scans were excluded.

### 2.4. Statistical Analysis

Linear regression and Pearson correlation analysis were performed to find out the correlation between visual acuity, central foveal point thickness, and ORT thickness. All the calculations and statistical analyses were performed using GraphPad Prism (version 6.0c).

## 3. Results

**Figure 1:**
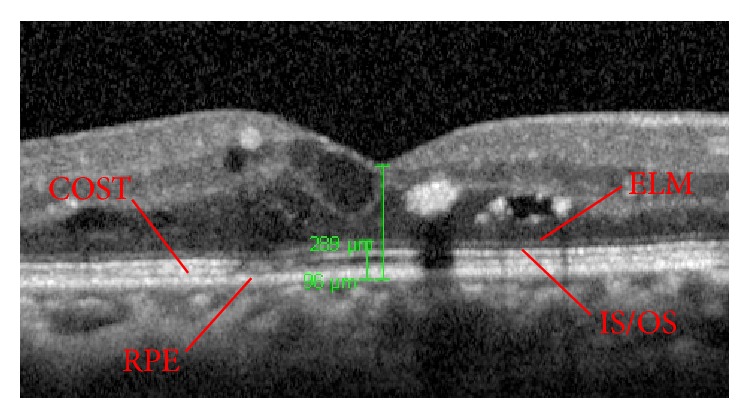
Representative ELM and RPE segmentation on optical coherence tomography (OCT) scan for measurement of central foveal point thickness and outer retinal layers (ORL) thickness. ELM, external limiting membrane. IS/OS, inner segment/outer segment. COST, cone outer segment tips. RPE, retinal pigment epithelium.

A total of 78 eligible patients were recruited. The mean age was 58.1 years (±11.5 years). 37 of the 78 patients were male (47.4%), therefore making almost 1 : 1 male : female ratio. Their mean spherical equivalent was −1.30 dioptres (±2.46 dioptres) and their mean Snellen visual acuity was 0.51 (±0.18). The mean central foveal point thickness of these 78 patients was 398.0 *μ*m (±74.3 *μ*m) whereas the mean outer retinal thickness was 115.7 *μ*m (±35.6 *μ*m). Representative example of ILM, ELM, and RPE segmentation and measurement is shown in Figure 1.

The correlation coefficient (*r*) and the square of correlation coefficient (*r* ^2^) between the central foveal point thickness and Snellen visual acuity were 0.34 and 0.12 in our study patients, respectively (*P* < 0.001). On the other hand, the correlation coefficient (*r*) and square of correlation coefficient (*r* ^2^) between ORL thickness and Snellen visual acuity were −0.65 and 0.42, respectively (*P* < 0.001).

## 4. Discussion

Diabetic macular edema is traditionally diagnosed clinically with biomicroscopic fundal examination. With the advancement of technology, OCT becomes an objective and highly reliable method in the assessment of such conditions. Besides measuring the actual macular thickening of the macula edema, newer generation OCT systems are capable of visualizing microretinal structures. Studies carried out worldwide have reported contradicting results regarding the correlation between central retinal thickness and visual acuity in DME [15–21]. Instead of using the total retinal thickness, Forooghian et al. measured the distance between photoreceptor inner segment/outer segment junction and RPE layer to approximate the length of photoreceptor outer segment (PROS) in patients with DME [14]. Forooghian's study showed that PROS length correlated better with patients' vision than macular thickness measurement. However, the intrasession repeatability of the PROS measurement with the self-developed OCT segmentation prototype software algorithm of Forooghian and his fellow colleagues was lower than the measurement of total macular retinal thickness with the Carl Zeiss OCT built-in software.

External limiting membrane is situated between cell nuclei of photoreceptors and their inner segments. The ORL thickness, as defined in this study as the distance between ELM and RPE, is therefore the sum of the length of photoreceptors inner segments and outer segments. It is known that photoreceptor outer segment contains disks filled with opsin, which is responsible for absorbing photons for later signal transduction. Therefore it is reasonable to deduce that if certain disease process damages photoreceptor and decreases the length of photoreceptor outer segment, vision would be compromised. This has already been proven to be true in DME by Forooghian and colleagues. On the other hand, the inner segment of photoreceptor is as important as the outer segment for cell functions because it is the reservoir of mitochondria; therefore it is responsible for the storage of ATP and thus for energy generation. Since both inner and outer segments of photoreceptors play an important role in the visual pathway, the change in ORL thickness should have important implications in visual potential. We believe this parameter can shed light on the overall health status of photoreceptors. Moreover, macular edema may be resolved but damage to photoreceptors does not; therefore a decrease in ORL thickness would be a more important visual prognostic factor than the total retinal thickness which may change over time.

In this study, we used Spectralis sd-OCT to quantify the foveal ORL thickness of our patients. We reported a relatively high correlation of the ORL thickness with visual acuity (*r* = 0.65, *P* < 0.001). In other words, we observed that the larger the ORL thickness is, the better the visual acuity would be. The correlation between total retinal thickness at the foveal center and visual acuity in our DME patients was not as good as the ORL thickness, with correlation coefficient of merely 0.34 (*P* < 0.001). This demonstrates good agreement between the findings of our study and Forooghian's study.

When compared with findings from other studies, we demonstrated that the ORL thickness and vision are more correlated than central retinal thickness and vision, although not as good as the correlation between PROS length and vision (Table 1). It is worth noting that ORL thickness is by definition longer than PROS length; therefore the same amount of systematic or random error in measurement would produce lower effect on the final result. For example, the mean PROS length on OCT is 32 *μ*m at fovea for DME patients [14] and the mean ORL thickness we obtained in this study is 115.7 *μ*m; if a random error of 5 *μ*m is generated by the OCT measurement, it would be 15.6% of the true PROS length but only 4.3% of the true ORL thickness. Moreover, there is only one study in the literature investigating the relationship of PROS length and vision in 30 DME patients. Provided that the repeatability of PROS length measurement is not excellent and the higher percentage error for any absolute error generated due to the shorter PROS length when compared to ORL thickness, it would be beneficial to conduct a larger scale study to compare the correlation between PROS length, ORL thickness and vision in order to further understand the strengths and shortcomings of the two different OCT parameters in the assessment of DME.

There are a number of limitations in our study and the assessment of ORL thickness. Patients with minimal DME without OCT scans done might have been missed during clinical assessment and therefore might not have been recruited in our study. Moreover, the exclusion of patients with subretinal fluid or disruption of ELM or RPE may also lead to selection bias.

It is time consuming to identify the ELM and RPE bands since all the segmentation was identified manually. It is also important to note that the measurement performed in this study was at the central foveal point rather than the measurement of central subfield thickness in most other studies; therefore our results of foveal thickness should not be directly compared to those from other studies.

ORL thickness of the foveal center may reflect a patient's visual potential at the point of fixation; however, activities of daily living such as reading rely highly on paracentral vision as well since it is necessary to locate the following word before one can move the point of fixation to the next word or next line with eye movement. ORL thickness measurement at foveal center does not adequately reflect this aspect of visual function. In order to address this problem, a new software algorithm could be developed to automatically locate the ELM segmentation after OCT scan is performed so as to calculate the ORL thickness in the central subfield instead of the foveal center.

Despite the promising results, ORL thickness cannot explain all the variations in vision since other factors such as macular ischaemia might play a role as well.

To conclude, we reported the use of ORL thickness as a novel OCT parameter in the assessment of DME patients and demonstrated that it is better correlated with vision than the total foveal point thickness. Further studies should be conducted to investigate the potential of using ORL thickness as a long term visual prognostic factor in DME patients.

## Figures and Tables

**Table 1:** Comparison of the results of the current study with other similar studies.

	Number of subjects	Parameters	*r* ^2^	*P* value
Present study	**78**	**ELM-RPE**	**0.42**	**<0.001**
Forooghian et al. [14], 2010	30	IS/OS-RPE	0.37–0.66^*^	<0.001
DRCR.net [8], 2007	251	CRT	0.27	<0.001
Ozdemir et al. [15], 2005	20	CRT	0.54	<0.001
Catier et al. [16], 2005	27	CRT	0.30	0.003
Bandello et al. [17], 2005	28	CRT	0.33	0.001
Laursen et al. [18], 2004	23	CRT	0.08	0.20
Massin et al. [19], 2003	15	CRT	0.13	0.19
Martidis et al. [20], 2002	16	CRT	0.15	0.14
Otani and Kishi [21], 2001	11	CRT	0.34	0.06

*r* ^2^, square of correlation coefficient. ELM, external limiting membrane. RPE, retinal pigment epithelium. IS/OS, inner segment/outer segment junction. CRT, central retinal thickness.

^*^Range of *r* ^2^ for macular grid, central subfield, and central point measurement.
